# The Radiologists’ Guide to Spinal Separation Surgery: What Does the Surgeon Want to Know?

**DOI:** 10.3390/diseases13100348

**Published:** 2025-10-18

**Authors:** Mohsin Khan, Labeeba Haq, Sai Niharika Gavvala, Petr Rehousek, Simon Hughes, Rajesh Botchu

**Affiliations:** 1 Department of Spinal Surgery, Royal Orthopedic Hospital, Birmingham B31 2AP, UK; 2 Department of Orthopedics, Royal Orthopedic Hospital, Birmingham B31 2AP, UK; 3 Department of Musculoskeletal Radiology, Royal Orthopedic Hospital, Birmingham B31 2AP, UK

**Keywords:** spinal neoplasms, spinal cord compression, palliative care, neurosurgical procedures, radiotherapy, stereotactic body, decompression, surgical, bone neoplasms, radiologic diagnosis, postoperative care

## Abstract

Spinal tumours are an uncommon but significant cause of pain, fractures, instability, and cord compression, leading to poor quality of life and mortality. Separation surgery is a rapidly advancing technique that has seen increased utilisation in the field of spinal oncology surgery. Separation surgery can be described as a resection technique that decompresses the spinal cord whilst creating an ablative target for high-dose stereotactic radiotherapy to achieve durable local control while minimising the risk of radiation myelopathy. This has facilitated the delivery of stereotactic radiotherapy, as well as created potential for use in managing primary bone tumours of the spine. From a radiology standpoint, optimal outcomes depend on meticulous preoperative characterisation of tumour volume and stability (e.g., ESCC grade and SINS), clear communication of anatomic constraints relevant to approach and fixation, and systematic postoperative surveillance to distinguish expected postoperative appearances from early recurrence or complications. We present our radiological experience and report recommendations while evaluating spinal oncology separation surgery.

## 1. Introduction

Spinal tumours, whether primary or secondary, can lead to pain, instability, neurological compromise, and eventual mortality, posing a significant surgical challenge [1]. En-bloc resection with clear margins is the definitive treatment for malignant primary spinal tumours, where the goal is preventing local recurrence and minimising distant metastatic disease. However, this intervention is associated with significant morbidity and may not always be anatomically feasible or appropriate [2]. Advancements in spinal separation surgery have sparked major interest. It is a technical procedure developed to control local tumour burden whilst permitting the safe and effective use of modern adjuvant radiotherapy techniques.

Separation surgery was introduced as a pragmatic alternative, designed to control tumour bulk while permitting the safe administration of high-dose stereotactic radiation [3,4]. This approach has become an essential technique in modern spinal oncology, particularly for metastatic spinal cord compression (MSCC) and radioresistant tumours.

This article explores the evolution and indications of spinal separation surgery in the management of spinal tumours. The aim is to provide the radiologist with a better understanding of preoperative surgical planning and postoperative surveillance to produce a comprehensive but clinically relevant report.

### 1.1. What Is Spinal Separation Surgery?

The concept of spinal separation surgery was introduced by Lyliana Angelov and Edward Benzel at the Cleveland Clinic, Ohio, as a pragmatic approach to manage metastatic and primary spinal tumours in anatomically complex or medically frail patients [1,2]. The principle lies in achieving circumferential decompression of the spinal cord, typically 2–3 mm, by resecting epidural tumour bulk while preserving vertebral column integrity. This manoeuvre creates a “safe margin” around the thecal sac, permitting the subsequent delivery of stereotactic radiosurgery (SRS) or stereotactic body radiotherapy (SBRT) at ablative doses, with significantly reduced risk of radiation-induced myelopathy [3,4,5].

Traditionally, the management of malignant spinal cord compression involved laminectomy followed by conventional external beam radiotherapy (cEBRT), a strategy shown to provide superior outcomes compared with cEBRT alone, particularly in radiosensitive tumours such as lymphoma, myeloma, and germinoma [6,7]. In these groups, cEBRT achieves local pain control in approximately 60–70% of patients with durable neurological improvement [8,9,10]. However, cEBRT is poorly effective against radioresistant histologies, including renal cell carcinoma, melanoma, and sarcomas, where long-term local control is typically less than 50% [11,12].

The advent of SRS and SBRT has dramatically altered the therapeutic landscape. These modalities deliver highly conformal, image-guided radiation with biologically effective doses (BED) up to threefold higher than cEBRT while sparing adjacent critical structures [3,5,13]. In spine oncology, this translates into the ability to ablate radioresistant tumour deposits, with local control rates exceeding 85–90% in modern series [14,15,16].

Despite these advantages, SBRT is limited by the radiation tolerance of the spinal cord and cauda equina. Exceeding a maximum cord dose of ~45–50 Gy (conventional fractionation equivalent) risks permanent neurological injury [17,18]. As many epidural tumours abut or compress the cord, full-dose delivery is impossible without unacceptable toxicity.

Separation surgery provides a technical solution to this problem. By resecting posterior vertebral elements (lamina, facets, pedicles) and mobilising the dura away from the tumour, a circumferential low-density margin of CSF or epidural fat is recreated. This allows SBRT planning to treat gross residual tumour with ablative doses while respecting spinal cord dose constraints [2,19]. Importantly, this operation does not aim for en-bloc or complete tumour excision but rather to enable durable local control through synergy with radiation therapy.

Alternative effective adjuvant radiotherapy techniques include intensity-modulated radiotherapy (IMRT), which permits highly conformal dose delivery with improved sparing of the spinal cord compared with conventional external beam radiotherapy (cEBRT). However, while IMRT reduces the collateral dose to surrounding tissues, it is generally less ablative than stereotactic body radiotherapy (SBRT) and is therefore reserved for scenarios where SBRT is contraindicated, for example, in patients with large or irregular target volumes, multifocal disease, or proximity to highly radiosensitive structures [20].

Another emerging modality is particle beam radiotherapy (PBT), including proton and carbon ion therapy, which leverages the Bragg peak to achieve a sharp dose fall-off beyond the target. This physical property enables radiation escalation beyond conventional spinal cord tolerance, making it particularly attractive for histologies such as sarcomas and chordomas that are otherwise resistant to photon-based therapies [21,22]. Early clinical series have reported encouraging local control rates in these settings, though access is limited by the high cost of treatment, technological complexity, and restricted global availability of proton/carbon ion centres [21,22].

Ultimately, the choice between SBRT, IMRT, and PBT is histology-dependent and must be individualised based on tumour radiosensitivity, anatomical constraints, prior radiation exposure, and available resources.

### 1.2. How Is Spinal Separation Surgery Performed?

Separation surgery can be performed through a range of approaches, including by transpedicular, costotransversectomy, lateral extracavitary, transthoracic, or retroperitoneal approaches, with the chosen strategy depending on tumour location, vertebral level, and degree of circumferential epidural involvement [13,14,15,23]. Our preferred method is the versatile single-stage transpedicular posterior approach, depending on tumour location [13,14,15]. This approach allows for both decompression and stabilisation through a single-stage posterior exposure [13,14]. Minimally invasive modifications of this technique have also been described, particularly in patients with limited disease or high perioperative risk [15,16,17].

The degree of circumferential decompression alongside tumour resection is extensive and often requires spinal column stabilisation. This is undertaken first with posterior pedicular instrumentation, two levels above and below the tumour, skipping the involved vertebrae [13]. Fixation can be achieved using free-hand, navigated, or minimally invasive techniques depending on surgeon preference [15,16,17]. This strategy maintains stability, reduces risk of construct failure, and prepares the spine for high-dose postoperative radiotherapy [15,16,17]. Special consideration must be given to implant selection. Compared to the traditional titanium implants, carbon-fiber/polyetheretherketone (PEEK) implants cause relatively less metallic susceptibility artefacts on CT and MRI, thereby improving radiotherapy dose accuracy as well as improving early detection of local tumour recurrence [18,19,20,21,22,24]. Several comparative studies suggest that carbon-based implants may improve the precision of stereotactic radiation delivery while maintaining equivalent mechanical safety [18,19,20,21,22,24].

The second stage involves posterolateral decompression via a laminectomy utilising a standard posterior midline exposure. The lateral component of the osseous decompression involves bilateral facetectomies and pediculotomies using a combination of a high-speed drill and Kerrison rongeurs. Bilateral pedicle resection provides access to the posterior vertebral body and anterior epidural space. This approach permits circumferential separation of the tumour from the dura. The posterior longitudinal ligament and Hoffman’s ligaments are typically divided, permitting separation of tumour from the thecal sac and the creation of a 2–3 mm margin of cerebrospinal fluid or epidural fat around the spinal cord [13,23,25]. This “separation” margin is the critical enabler of safe, high-dose stereotactic radiosurgery. Residual tumour within the vertebral body may also be partially resected. If >50% of the vertebral body is removed, reconstruction is required to prevent collapse and instrumentation failure. This is commonly achieved using an expandable interbody cage—titanium- or PEEK-based—inserted into the corpectomy cavity and supplemented with bone graft material [26].

### 1.3. What Are the Tumours Appropriate for Spinal Separation Surgery?

#### 1.3.1. Metastatic Spinal Cord Compression

**Figure 1:**
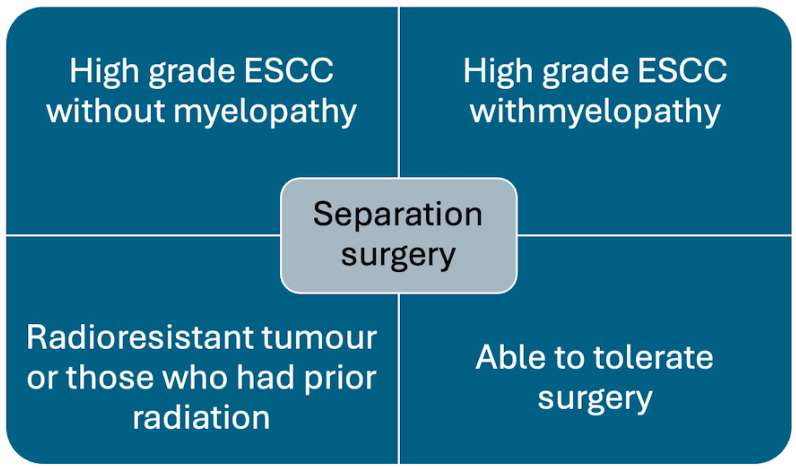
Chart showing indications for separation surgery.

Separation surgery is being increasingly utilised to treat metastatic spinal cord compression (MSCC) [23]. It is most effective and recommended in patients with tumours causing high-grade epidural spinal cord compression (ESCC) and tumours that are radioresistant, e.g., renal cell carcinoma, melanoma, sarcomas, and prior history of radiotherapy, where further cEBRT is not feasible (Figure 1) [3,4].

The introduction of separation surgery has allowed for durable local control even in this difficult cohort. In the first large outcome analysis, Laufer et al. reported results in 186 patients undergoing separation surgery followed by adjuvant SRS for MSCC due to predominantly radioresistant tumours [25]. Local control was excellent, with a 1-year cumulative local recurrence rate of 9% for patients treated with single-fraction high-dose SRS and 4% in patients receiving high-dose adjuvant hypofractionated SRS.

Similar outcomes have been reported for MSCC caused by histology-specific tumours. The 2-year cumulative incidence of local recurrence was 4.6% for 90 patients with renal cell carcinoma [27] and 5.4% for 103 patients with non-small-cell lung cancer [28].

These results underscore the role of separation surgery as a cornerstone of hybrid therapy in MSCC, particularly where durable control with cEBRT alone would be improbable.

#### 1.3.2. Spinal Sarcomas

For primary sarcomas of the spine, en-bloc resection remains the gold standard when feasible [1,2]. However, anatomical complexity and morbidity often preclude such radical procedures. In this setting, separation surgery combined with modern radiotherapy has been explored as a definitive alternative. Kanda et al. reported the successful treatment of a primary osteosarcoma of the spine using separation surgery followed by adjuvant IMRT, where en-bloc excision was not technically feasible [29]. Similarly, Kanda et al. described a case of high-grade spinal myxofibrosarcoma treated with separation surgery plus postoperative IMRT, achieving durable local control [30].

These reports suggest that separation surgery can serve as an organ-preserving, function-preserving alternative in select sarcomas when en-bloc margins cannot be achieved.

Spinal chordomas are the archetypal radioresistant tumour but have been shown to respond favourably to surgery and adjuvant single-fraction SRT [31]. Lockney et al. reported excellent local control on treating 12 spinal chordoma patients with separation surgery and adjuvant SRT [32]. All patients received five or fewer fractions of high-dose SRT within 4 months of separation surgery. Actuarial overall survival at 2 years was an estimated 77.6 months (95% CI 52–103.3 months). Local tumour control rate was 80% when separation surgery with adjuvant SRT was used as the definitive procedure compared to 57.1% when used as a salvage procedure alone.

Jung et al. demonstrated that chordomas respond favourably to single-fraction spine SBRT, with meaningful local control [31].

In a series by Lockney et al., 12 patients with spinal chordomas underwent separation surgery followed by high-dose SBRT (≤5 fractions) within 4 months [32]. The results showed an actuarial overall survival at 2 years of 77.6 months (95% CI, 52–103.3 months) and a local control rate of 80% when used as a definitive strategy, compared with 57.1% when used as salvage therapy.

This highlights that in selected patients with chordoma—particularly those unfit for or unsuitable for en-bloc resection—separation surgery combined with SBRT can provide durable disease control.

#### 1.3.3. Adjuvant Radiotherapy Techniques

Adjuvant radiotherapy in spinal oncology is administered to cater mainly two purposes: (1) pain relief and (2) local disease control to prevent or even improve neuronal compromise. The choice of radiotherapy technique is case-specific and depends upon tumour histology, surgical margins, prior treatment, and patient performance status.

#### 1.3.4. Conventional External Beam Radiotherapy (EBRT)

Conventional EBRT remains a cornerstone for radiosensitive tumours such as lymphoma, multiple myeloma, and germinoma. It employs photon-based delivery and offers reliable long-term disease control in this subgroup [4,10]. Fractionation regimens vary, but common schedules include single-fraction (8 Gy) or multifraction regimens (e.g., 20 Gy in 5 fractions) [4]. Although single-fraction EBRT is cheap and convenient, it may be associated with poor results and may necessitate repetition [4,9,33]. However, for radioresistant tumours, the long-term local disease is consistently less than 50% [34,35].

Single-fraction EBRT is cost-effective and convenient, but its efficacy in terms of durable tumour control and pain relief is inferior to multifraction approaches and often necessitates retreatment [4,9,33]. Importantly, EBRT provides suboptimal outcomes in radioresistant tumours, with long-term local control rates consistently reported as <50% in histologies such as renal cell carcinoma and sarcoma [34,35].

#### 1.3.5. Stereotactic Body Radiotherapy (SBRT) and Stereotactic Radiosurgery (SRS)

SRS and SBRT have revolutionised the management of spinal tumours, particularly those resistant to cEBRT. These techniques are approximately three times more biologically effective than cEBRT. Spinal SRS is delivered in a single fraction, whereas SBRT is delivered in two to five fractions [4,6].

Both procedures are labour-intensive and require a detailed anatomical evaluation to visualise the gross tumour volume as well as its proximity to the spinal cord. A pretreatment MRI scan with or without a spinal myelogram must be obtained, ensuring adequate immobilisation to minimise target motion to <1 mm, facilitated by computer-based planning and image guidance [4,6,36]. Spinal SBRT is very precise, with irradiation margins of approximately 0–3 mm beyond the target zone compared to 7–10 mm using conventional EBRT [37,38,39]. Adverse effects of SBRT are rare but include fatigue, pain flare reaction, acute dermatitis, oesophagitis, fistula formation, symptomatic radiation, and myelopathy [6]. Vertebral insufficiency fractures are late but well-recognised complications and have a reported incidence rate of approximately 14% [40].

#### 1.3.6. Particle Beam Therapy (PBT)

Particle beam radiotherapy utilises either protons or carbon ions. It is underpinned by the ‘Bragg peak’ principle, which describes that maximal ionisation, or irradiation, will occur immediately before the particles come to rest. Essentially, this allows for focused high-dose radiation to be delivered accurately at a pre-determined depth with minimal damage to soft-tissues beyond this depth [6,41]. The maximal dose of radiation tolerated by the spinal cord, before becoming neurotoxic, is limited to 45–50 Gy, which is suboptimal in controlling sarcomas [42,43]. PBT circumvents this and is currently being employed as a postoperative adjuvant treatment for spinal sarcomas in selected patients, with promising early results [44,45].

A phase II clinical trial has reported outcomes on postoperative high-dose photon/proton radiotherapy for non-metastatic spinal sarcomas in 50 patients, with a median follow-up 7.3 years [45]. Patients were aged over 16 years with a Karnofsky Performance Status (KPS) greater than 70, excluding loss of function secondary to the local tumour growth. Primary disease was present in 36 patients (72%), and 14 patients (28%) had locally recurrent disease. Depending on histology (58% chordomas and 28% chondrosarcomas), the cumulative radiation dose delivered ranged from 59.4 Gy to 77.4 Gy. Overall survival was 84% at 5 years and 65% at 8 years. Recurrence-free survival was 64% at 5 years and 52% at 8 years. Local control in 36 patients treated for primary disease was 81% at 5 years and 74% at 8 years. Distant metastases were identified in four patients with chordoma and three patients with chondrosarcoma. A total of 12 patients sustained a radiation-related complication, which comprised 4 sacral insufficiency fractures, 2 sacral neuropathies, and 2 erectile dysfunctions. No spinal cord injuries were noted. The actuarial risks for radiation-related complications (any grade I-IV) were 16% at 5 years and 19% at 8 years.

Interestingly, local failure was reported in 5 of 16 patients (31%) with spine stabilisation (metallic implants) compared to 4 out of 34 patients (12%) without hardware [44]. This may represent a larger residual disease volume in patients requiring instrumentation. However, it is more likely that artifact caused by metallic implants impedes accurate visualisation of tumour tissue, which has led to increased demand for radiolucent carbon-fiber-reinforced polyether ether ketone (CFR-PEEK) implants [46,47,48,49].

The main disadvantage of PBT is hampered by its extreme cost, which limits its global availability. Also, there is lack of long-term data in evaluating efficacy and safety in spinal separation surgery, which is currently under study [45,50].

## 2. Case Examples

### 2.1. Case 1: Cervical Chordoma

**Figure 2:**
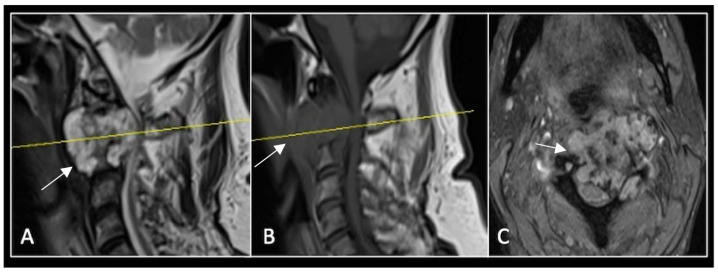
MRI demonstrating a destructive lobular lesion localised to the C2 vertebra (chordoma) (arrow). There is significant left-sided extension into the paravertebral tissues laterally and epidural space centrally with indentation of the cord. The yellow line on the sagittal images denotes the level at which the corresponding axial section was obtained. (**A**) T2-W sagittal; (**B**) T1W sagittal; (**C**) T2W axial.

**Figure 3:**
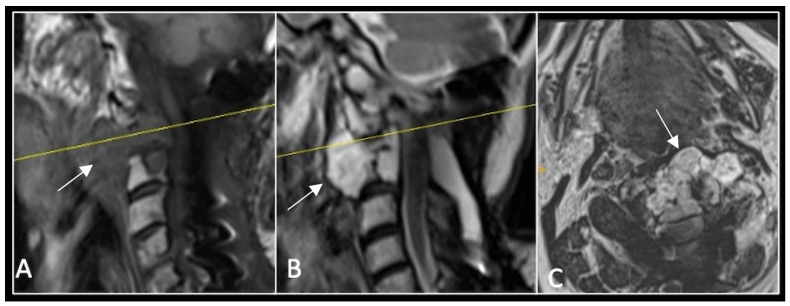
RI post-separation surgery showing reduction in tumour (arrow) dimensions and circumferential decompression. The posterior elements have been removed, and a posterior occipitocervical stablilsation has been performed. En-bloc resection would have necessitated sacrificing the left C2 nerve root and vertebral artery. The yellow line on the sagittal images indicates the level of the corresponding axial section. (**A**) T2W sagittal; (**B**) T1W sagittal; (**C**) T2-W axial.

A 73-year-old male presented with an eight-month history of neck pain that was exacerbated on lying flat. He denied any symptoms of myelopathy and radiculopathy. He did not have any constitutional symptoms of malignancy. Past medical history included hypertension, renal cysts, and an ascending thoracic aneurysm. Examination was unremarkable. Diagnostic whole-spine MRI imaging demonstrated a large lobular mass localised to the cervical spine (Figure 2). Biopsy confirmed a conventional chordoma, and he underwent separation surgery followed by adjuvant proton beam therapy. Postoperative surveillance imaging at 12 months demonstrated stable disease (Figure 3).

### 2.2. Case 2: Sacral Chordoma

**Figure 4:**
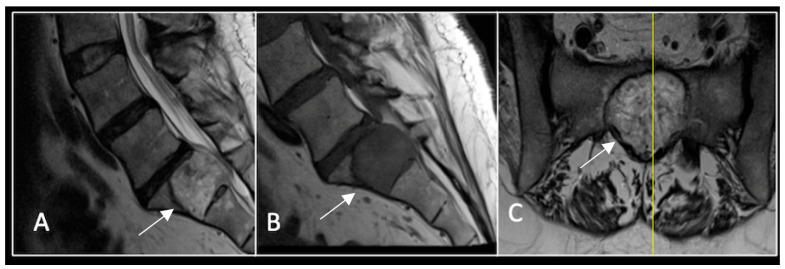
MRI demonstrating a destructive lobular sacral mass (arrow) with invasion of the S1 vertebral body and complete compression of the cauda equina neural elements. The yellow line on the axial image represents the plane of the corresponding axial section. (**A**) T2W sagittal; (**B**) T1W sagittal; (**C**) T2W axial.

**Figure 5:**
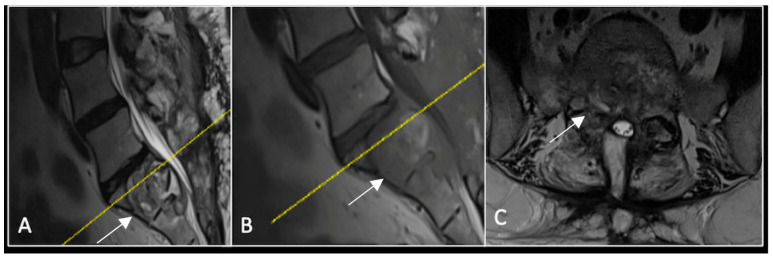
MRI imaging following separation surgery. (**A**) T2W sagittal; (**B**) T1W sagittal; (**C**) T2W axial. Imaging shows removal of posterior lumbosacral elements with residual disease (arrow) within the S1 vertebral body but significant reduction in tumour dimensions. The yellow line on the sagittal images indicates the level at which the axial image was obtained.

**Figure 6:**
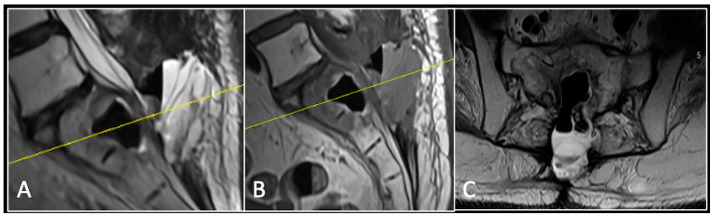
MRI imaging following revision debulking surgery. (**A**) T2W sagittal; (**B**) T1W sagittal; (**C**) T2-weighted axial. Imaging shows an air cavity within the S1 vertebral body where tumour has been resected and fluid within the epidural space that is displacing the thecal sac posteriorly. The yellow line on the sagittal images denotes the level corresponding to the axial section.

This 63-year-old male presented with sacral pain and right-sided S1 radiculopathy. He was subsequently referred to our institution after diagnostic imaging revealed a destructive lesion within the S1 vertebra (Figure 4). The patient developed symptoms of acute cauda equina syndrome, for which we recommended an emergent surgical decompression to be performed at the referring hospital. Biopsy confirmed a conventional chordoma. The patient chose to undergo separation surgery, given the significant morbidity associated with an en-bloc sacral resection, including sacrifice of the neural structures innervating his bladder and bowel. He received adjuvant proton beam therapy, and 6-monthly surveillance imaging revealed stable disease (Figure 5). Unfortunately, 12 months post-separation surgery, there was evidence of tumour progression with epidural extension and symptomatic deterioration in bladder function, requiring revision surgical debulking (Figure 6).

### 2.3. Case 3: Cervical Chordoma

**Figure 7:**
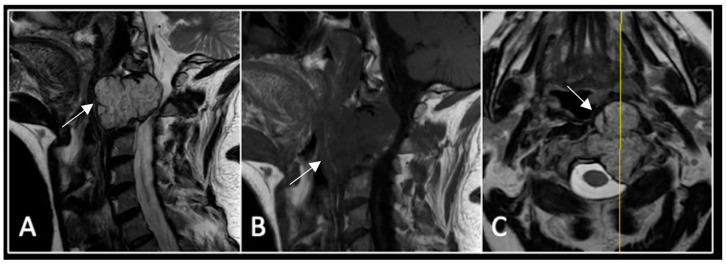
The MRI demonstrates a left-sided lobular mass (arrow) with invasion of the C2/3 vertebral body and epidural extension, but no cord compression. The yellow line on the axial image indicates the axial slice level. (**A**) T2W sagittal; (**B**) T1W sagittal; (**C**) T2W axial.

**Figure 8:**
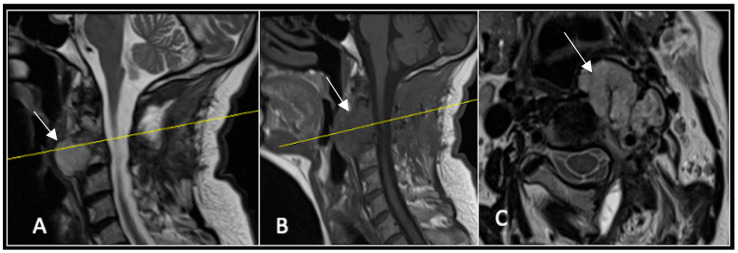
MRI demonstrating a left-sided posterior approach to the cervical spine with significant reduction in residual gross tumour volume (arrow) and circumferential decompression of the spinal cord. The yellow line on the sagittal images denotes the level corresponding to the axial section. (**A**) T2-weighted sagittal; (**B**) T1-weighted sagittal; (**C**) T2-weighted axial.

**Figure 9:**
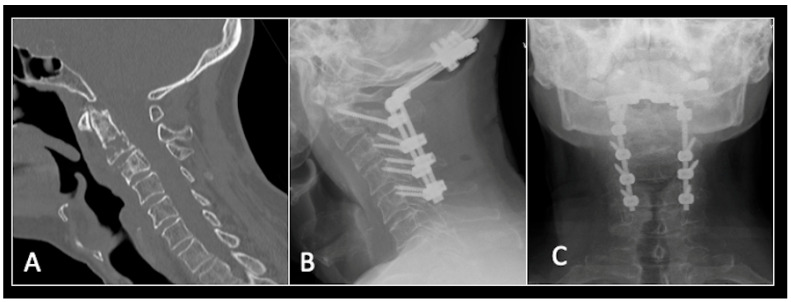
(**A**) Sagittal CT imaging showing pathological fracture of C2 vertebral body. (**B**,**C**) Posterior occipitocervical stabilisation on lateral (**B**) and AP (**C**) radiographs, respectively.

**Figure 10:**
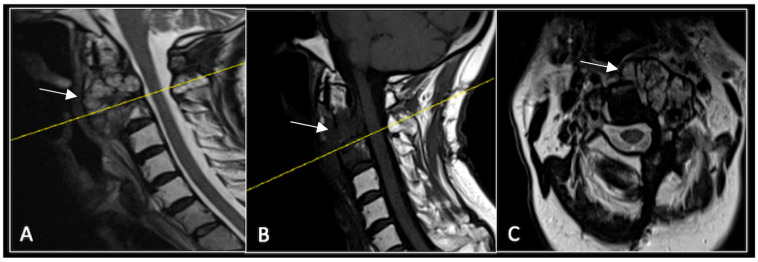
RI demonstrating no tumour (arrow) progression with wide circumferential margins around the spinal cord. The yellow line on the sagittal images represents the level of the axial plane. (**A**) T2W sagittal; (**B**) T1W sagittal; (**C**) T2W axial.

This 68-year-old male was referred to our institution with a 4 cm cervical mass localised to C2/3 following investigations for left-ear congestion and altered hearing (Figure 7). A CT-guided biopsy confirmed a conventional chordoma. The patient underwent separation surgery with adjuvant proton beam therapy. There was no evidence of disease progression on 3-monthly surveillance imaging (Figure 8). Approximately 12 months following separation surgery, the patient presented with acute onset neck pain. A C2 odontoid peg fracture was diagnosed requiring a posterior occipitocervical stabilisation procedure (Figure 9). Pre-stabilisation MRI imaging confirmed no tumour progression (Figure 10).

### 2.4. Case 4: Lumbar Chordoma

**Figure 11:**
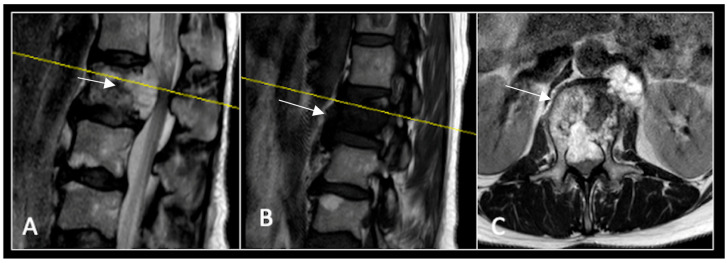
RI demonstrating a lobulated destructive lesion (arrow) with significant epidural extension and compression at L2. The yellow line on the sagittal images denotes the level of the axial image. (**A**) T2W sagittal; (**B**) T1W sagittal; (**C**) T2W axial.

**Figure 12:**
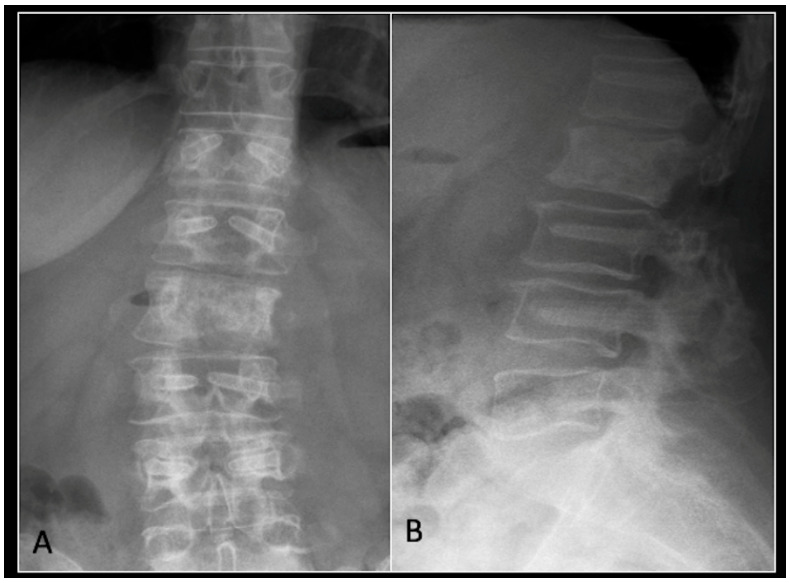
(**A**) demonstrates an anteroposterior view, and (**B**) illustrates a lateral standing radiograph of the lumbosacral spine demonstrating carbon-based posterior instrumentation.

**Figure 13:**
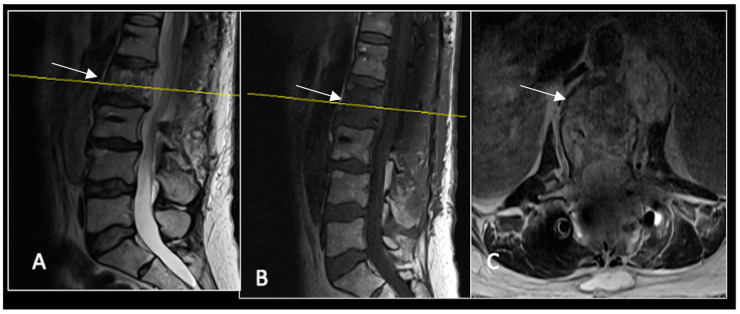
A 6-month postoperative surveillance MRI shows no obvious evidence of residual tumour (arrow) disease progression, but there is significant artefact. The yellow line on the sagittal images denotes the level of the axial image. (**A**) T2W sagittal; (**B**) T1W sagittal; (**C**) T2W axial.

**Figure 14:**
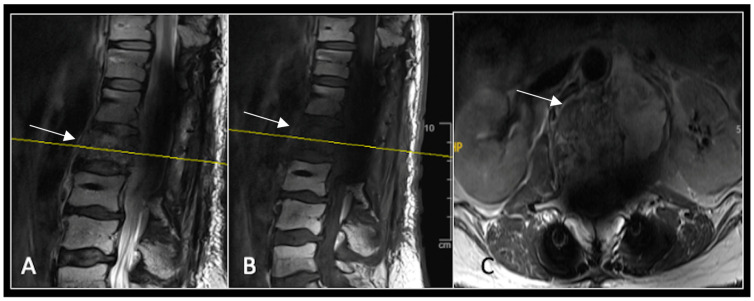
A 12-month postoperative surveillance MRI shows no evidence of residual tumour (arrow) disease progression. The severity of artefact is reduced. The yellow line on the sagittal images denotes the level of the axial image. (**A**) T2W sagittal; (**B**) T1W sagittal; (**C**) T2W axial.

This 56-year-old lady was diagnosed with a biopsy-confirmed L2 chordoma (Figure 11). She underwent posterior decompressive separation surgery and instrumental stabilisation T12-L4 using carbon-based implants (Figure 12). Surveillance imaging at 6 months and 12 months post-separation did not show any progression of residual tumour disease (Figure 13 and Figure 14).

### 2.5. The Radiological Report

Understanding the goals of spinal separation surgery helps the radiologist plan a clinically relevant report of imaging used for preoperative surgical planning and imaging used for surveillance. A comprehensive system for reporting en-bloc resection of spinal tumours has already been described [51]. This includes tumour location and dimensions, involvement of neural and vascular structures, and stability of the vertebral column. We recommend the following approach when reporting on the nuances of separation spinal surgery.

### 2.6. Tumour Location and Dimensions

The vertebral levels involving tumour tissue should be stated, including the presence of transitional vertebrae and the methodology used to delineate vertebral segmentation (i.e., counting vertebrae cranially from the sacrum or caudally from C2). Measuring longitudinal and transverse tumour dimensions serves a dual purpose. It aids with preoperative surgical planning in determining circumferential tumour resection margins as well as monitoring disease recurrence on surveillance imaging. Longitudinally, this includes the number of vertebral and intervertebral discs requiring partial resection. In the transverse plane, the degree of tumour extension into local osseous and soft-tissue structures must be noted.

### 2.7. Neuraland Vascular Structures

It is crucial to describe the extent of tumour invasion into the spinal canal and whether there is encasement of the segmental nerves. One of the principles of oncological surgery is to ensure tumour resection without contamination. However, this may entail sacrificing neural structures, resulting in profound functional loss, which must be discussed with the patient and the wider multidisciplinary team. Separation spinal surgery with adjuvant radiotherapy may be selected in cases of revision surgery or may even be preferred by the patient as the primary treatment modality.

Tumour extension into the vertebral arteries may preclude en-bloc resection in the cervical spine. Even in cases where spinal separation surgery is indicated it would be recommended to perform a computed tomography (CT) angiography to assess for anatomical variations and vessel dominance within the circle of Willis vasculature.

### 2.8. Vertebral Column Stability

This is assessed on preoperative CT imaging, especially when planning the circumferential extent of osseous tumour resection and the number of vertebral levels involved.

### 2.9. Surveillance and Disease Progression

One must remember that residual tumour tissue will remain following spinal separation surgery. One of the facets of radiological reporting is to recognise tumour progression and whether further debulking surgery may be indicated. This, of course, is dependent on patient symptomology (pain and neurological function) and risk factors for local progression (tumour type, extent of surgical decompression, and dose of adjuvant radiotherapy). At our institution, spinal sarcoma radiological surveillance is performed every 3 months for the first 2 years after surgery, then every 6 months for a further 3 years, and then annually till the patient is 10-years post-procedure.

### 2.10. Challenges of Surveillance Imaging

The SPINO (spine response assessment inneuro-oncology) guidelines recommend MRI as the imaging modality of choice for tumour surveillance [52,53,54]. However, there is no consensus on what measurement thresholds would constitute tumour progression [54]. Interpretation is extremely challenging and requires clinical context. It is unclear what signal changes on a T1-weighted non-contrast-enhanced scan best characterise tumour regression or progression [52]. Furthermore, signal changes on both T1- and T2-weighted sequences may represent a combination of osteoradionecrosis, fibrosis, and vertebral insufficiency fracture rather than true tumour progression [52]. In addition, there is limited data on pseuodo-progession, which is an imaging-based phenomenon defined as a transient increase in apparent tumour size following SBRT [52]. Positron emission tomography (PET) and spinal perfusion imaging may provide a more accurate response prediction, but routine utilisation in tumour surveillance is yet to be determined [54,55,56].

## 3. Conclusions

Spinal separation surgery, when combined with modern adjuvant radiotherapy techniques such as SBRT and particle beam therapy, represents a significant paradigm shift in the management of spinal tumours. This hybrid approach enables durable local control even in traditionally radioresistant histologies while minimising morbidity compared with en-bloc resection.

Although early patient-reported outcomes, including pain relief, neurological preservation, and local disease control, are promising, longer-term cohort studies are required to fully elucidate tumour progression patterns, especially radiologically. Table 1 summarises the five major domains a radiologist must focus upon while reporting, which are useful for preoperative surgical planning or for postoperative tumour surveillance.

For radiologists, accurate interpretation of pre- and postoperative imaging is pivotal, not only to guide surgical planning and radiotherapy targeting but also to avoid misinterpretation of expected postoperative changes as recurrence.

## Figures and Tables

**Table 1:** The template of components that should be mentioned in the radiologist’s report.

Template
Tumour location and dimensions
2. Neural structure involvement
3. Vascular structure involvement
4. Stability
5. Residual disease progression

## Data Availability

Data are available to share on request.
